# Environmental Risk and Management of Iron Tailings in Road Subgrade

**DOI:** 10.3390/toxics13070603

**Published:** 2025-07-17

**Authors:** Xiaowei Xu, Dapeng Zhang, Jie Cao, Chaoyue Wu, Yi Wang, Jing Hua, Zehua Zhao, Jun Zhang, Qi Yu

**Affiliations:** 1 Nanjing Institute of Environmental Science, Ministry of Ecology and Environment of China, Nanjing 210042, China; xuxiaowei@nies.org (X.X.); zhangdapeng@nies.org (D.Z.); caojie@nies.org (J.C.); wuchaoyue@nies.org (C.W.); wangyi@nies.org (Y.W.); huajing@nies.org (J.H.); 2 Department of Environmental Science, School of Environmental Science and Engineering, Suzhou University of Science and Technology, Suzhou 215009, China; yuqi@usts.edu.cn

**Keywords:** iron tailings, resource utilization, heavy metal, risk management

## Abstract

The utilization of iron tailings in road construction poses significant environmental risks due to the complex release mechanisms of pollutants and varying regional conditions. This study integrates an exponential decay model with an instantaneous pollutant transport model, employing Monte Carlo simulations to assess risks and regional characteristics. Results show high Potential Hazard Indices (PHIs) for arsenic, manganese, barium, nickel, and lead, with PHI values between 4.2 and 22.7. Simulations indicate that manganese and nickel concentrations may exceed groundwater standards, particularly in humid areas. The study recommends controlling the iron tailings mixing ratio based on climate, suggesting limits of 35% in humid, 60% in semi-humid, and more lenient ratios in arid and semi-arid regions. It also underscores the need for improved risk assessment methodologies and region-specific management strategies at the national level.

## 1. Introduction

Based on authoritative data from the Annual Report on the Prevention and Control of Solid Waste Pollution published by the Ministry of Ecology and Environment, the annual increase in tailings from the ferrous metal mining and processing industry in China has exceeded 440 million tons, while the comprehensive utilization rate remains low, hovering at around 23.4% [1]. This highlights the severe contradiction between resource utilization and environmental protection [2,3]. Iron tailings have complex compositions, being rich in elements such as silicon, aluminum, and calcium, while also containing potentially harmful substances such as cobalt, nickel, and arsenic [4]. If not properly disposed of, they will not only occupy a large amount of land resources but also easily induce a series of environmental problems such as soil ecological degradation and groundwater pollution, directly affecting public health and safety [5]. The Chinese “Guiding Opinions on the Comprehensive Utilization of Bulk Solid Wastes During the 14th Five-Year Plan Period” strategically proposes to increase the comprehensive utilization rate of bulk solid wastes to 60% and strives to reduce and harmlessly treat existing solid waste inventories. In this context, the efficient resource utilization of iron tailings has become a core issue and urgent challenge for the green transformation and high-quality development of the ferrous metal mining and processing industry [3,5].

The resourceful conversion of iron tailings has been a research focus in related fields in recent years [1,6]. Iron tailings, primarily composed of elements such as silicon, aluminum, and calcium, can be utilized in building materials, such as non-fired bricks and concrete, and as filling materials in mine pits and roads [4]. Research by Lang et al. [7] evaluated the mechanical properties of road-building materials prepared by mixing iron ore tailings with soil. The results showed that by adjusting the proportions of iron ore tailings added, the compressive strength of the road-building materials could meet the required standards. Liu et al. [4] investigated the preparation of sustainable cement clinker and environmentally friendly cementitious materials using iron ore tailings. Their findings revealed that, while ensuring the material properties met the required standards, the blending ratio of iron tailings could reach up to 60%. Almeida et al. [8] conducted a study on the environmental risks associated with iron tailings, which indicated that iron tailings significantly increased the levels of heavy metals such as copper in the surrounding soil environment. Wang et al. [3] performed a feasibility study on the preparation of sintered bricks from iron tailings. The results demonstrated that iron tailings contained multiple types of heavy metals with varying concentrations, among which copper, zinc, and lead were present at higher levels. Increasing the sintering temperature could effectively reduce the mobility of these heavy metals.

The current risk assessments for solid waste resource utilization predominantly rely on steady-state models based on traditional leaching experiments, which assess risk by comparing pollutant leaching concentrations to groundwater quality standards. However, these models overlook the unsteady-state release and complex migration processes of pollutants from materials used over the long term [6,9,10]. Additionally, many assessments use uniform national parameters that fail to account for regional meteorological differences, particularly variations in precipitation patterns, leading to incomplete risk evaluations. For instance, a study by Sun et al. [11] found that in road applications using fly ash as a road base material, exposure concentrations and environmental risks were positively correlated with precipitation levels, both increasing significantly with higher rainfall. Li et al. [12] emphasized in their environmental risk assessment of red mud-modified phosphogypsum self-leveling mortar that it was crucial to consider the migration and transformation characteristics of contaminants such as chromium and cadmium in the environment. Bai et al. [13] investigated permeable concrete paving bricks made from electrolytic manganese residue, red mud, and fly ash, demonstrating that optimizing production processes could ensure that leaching toxicity levels of nickel, copper, zinc, and manganese met regulatory standards. They highlighted the importance of adopting dynamic models to simulate the long-term release and transformation behaviors of heavy metals, thereby enhancing the accuracy of risk assessments.

To address these issues, this study integrates source strength exponential decay with instantaneous pollutant migration models and combines Monte Carlo simulation methods to reveal the probability and temporal evolution characteristics of shallow groundwater contamination under scenarios of iron tailings used in road construction in arid, semi-arid, semi-humid, and humid regions. Additionally, this study derives the maximum blending ratio of iron tailings in subgrade materials, aiming to provide a reference for risk assessments as well as regionally differentiated management and control of iron tailings used in road constructions.

## 2. Materials and Methods

### 2.1. Sample Collection and Analytical Detection

This study focuses on an illegal dumping event of approximately 140,000 tons of iron tailings into a borrow pit in the northeastern plains of the Jiangsu Province, China. The study area is characterized by a warm temperate humid monsoon climate, with an annual rainfall of 900 mm.

In accordance with the site layout and sampling methods specified in HJ 298-2019 [14], a total of 100 samples were collected. These samples were then quantitatively analyzed using the pretreatment and detection methods stipulated in GB 5085.3-2007 [15] and GB 5085.6-2007 [16].

### 2.2. Characterization of Pollutant Release

During the utilization of iron tailings in road construction, pollutants are leached and released into the underlying soil and groundwater environment by rainfall infiltration [17]. This study employs an exponential decay source model to describe the change in the pollutant release concentration within the source strength, represented by Equations (1) and (2) [18] as follows:

(1) $$C_{t}=C_{0}\times e^{-\lambda t}$$

(2) $$\lambda =\frac{\alpha C_{j}}{df\rho C_{sw}}$$

where $C_{t}$ represents the release concentration of pollutants in the source strength at any time *t* in mg/L; $C_{0}$ is the initial concentration of pollutants in the leachate in mg/L; α is the groundwater infiltration rate in m/a; *d* is the thickness of the road base layer in m; ρ is the density of the road base layer in kg/L; $C_{j}$ is the leaching concentration of pollutants in mg/L; *f* is the volume fraction of iron tailings in the road base layer and is dimensionless; ρ is the density of the roadbase layer in kg/L; $C_{j}$ is the leachate concentration of the contaminants obtained using the NEN 7371 [19] method in mg/L; and $C_{sw}$ is the maximum effective release amount of pollutants in mg/kg.

The Dutch standard NEN 7371 leaching method [20] is used to determine the maximum effective release of inorganic components from wastes and materials under extreme/adverse environmental conditions. This experimental method has been widely adopted by the EU countries to assess the environmental safety of building materials. Based on the NEN 7371 protocol, a two-stage leaching experiment was conducted as follows: First, the pH of the leaching solution was stabilized between 6.5 and 7.5 for 3 h and then, the pH was maintained between 3.5 and 4.5 for another 3 h. The liquid-to-solid ratio was set at 50:1 (L/kg) for both stages. Equal volumes of the leachates obtained from each stage were thoroughly mixed before their concentrations were analyzed.

### 2.3. Characterization of Pollutant Migration and Diffusion

In this study, the most adverse exposure scenario is selected for diffusion characterization, where the iron tailings in the road base material are in direct contact with the soil, and there is no rain shelter above the road base material. Under the influence of rainfall, pollutants in the road base material will be leached out with rainwater and released into the environment [21]. Through diffusion and infiltration, these pollutants will penetrate the saturated layer and migrate with the groundwater flow, spreading into observation wells, and thereby affecting the environment and human health. Throughout the process of pollutant release from the road base material, the concentration of the pollutants is diluted and reduced by diffusion and migration. The natural attenuation process can be divided into two main steps [21]: the first step is the vertical process (LF) of the potentially toxic elements in the road base material being leached out into groundwater by rainfall infiltration, and the second step is the horizontal process (DAF) of the pollutants migrating, diluting, and diffusing into observation wells with the groundwater flow after entering the groundwater. Based on the exposure pathways of the characteristic pollutants in the road base material, the concentration of the pollutants in the road base material, after being diluted and attenuated, can be expressed in terms of the concentration of pollutants in the groundwater using Equation (3) [21]:

(3) $$c_{gw}=c_{w}\times LF\times DAF$$

where $c_{gw}$ represents the concentration of pollutants in the groundwater at the observation well in mg/L; $c_{w}$ represents the content of pollutants released from the pollution source in mg/kg; *LF* is the soil leaching and dilution factor in kg/L; and *DAF* is the groundwater dilution and attenuation coefficient, which is dimensionless.

#### 2.3.1. Soil Leaching and Dilution Factor (LF)

To calculate the leaching and dilution factor (LF) for the pollutants in road base materials, a typical heavy metal attenuation and diffusion model should be established that can simulate the actual use scenario of road base materials. Pollutants undergo adsorption–desorption, chemical reactions, as well as hydrodynamic migration and molecular diffusion in the unsaturated soil layer [21]. However, in the long term, both adsorption and chemical reactions have certain capacities. For scenarios with high-concentration sources such as from the utilization of solid waste in road construction, from a conservative assessment perspective, adsorption and reactions can be neglected. Based on the leaching factor calculation model for pollutants migrating into groundwater specified in HJ 25.3-2019 [22], and considering various influencing factors such as soil density, permeability, the pollutant partition coefficient, the thickness of the mixing zone, and the moisture content of the unsaturated zone, the dilution factor LF during the leaching process of metal pollutants from road base materials migrating and diffusing into groundwater is calculated using Equations (4) to (6) [23]:

(4) $$LF=\frac{{LF}_{spw-gw}}{K_{sw}}$$

(5) $${LF}_{spw-gw}=\frac{1}{1+\frac{U_{gw}\;\times \;\delta_{gw}}{I\;\times \;W}}$$

(6) $$K_{sw}=\frac{\theta_{ws}+K_{d}\times \rho_{b}+H\times \theta_{as}}{\rho_{b}}$$

where *LF* represents the leaching factor for pollutants migrating from road base materials into groundwater in kg/L; ${LF}_{spw-gw}$ is the leaching factor for pollutants migrating from soil pore water into groundwater, which is dimensionless; $K_{sw}$ is the partition coefficient of pollutants between soil and water in m^3^/g; $U_{gw}$ is the Darcy velocity of groundwater in m/a; $\delta_{gw}$ is the thickness of the groundwater mixing zone in m; *I* is the infiltration rate of water in the soil in m/a; *W* is the width of the road in m; $K_{d}$ is the partition coefficient of pollutants between the soil solid phase and water in m^3^/g; $\theta_{ws}$ is the volumetric ratio of pore water in unsaturated soil, which is dimensionless; $\theta_{as}$ is the volumetric ratio of pore air in unsaturated soil, which is dimensionless; $\rho_{b}$ is the bulk density of soil in kg/L; and *H* is the Henry’s constant for the pollutant, which is dimensionless.

#### 2.3.2. Groundwater Dilution and Attenuation Factor (DAF)

The dilution and attenuation of pollutants in groundwater are controlled by processes such as the dilution effect of groundwater flow (i.e., convection) and the attenuation effect of the subsurface medium (i.e., degradation, dispersion, and retardation) [24]. In homogeneous and isotropic soil–water systems, the transport process as well as the dilution and attenuation effects can be modeled using the one-dimensional advective–dispersive equation, as shown in Equation (7) [25]:

(7) $$\frac{\partial C}{\partial t}=D_{L}\frac{\partial^{2}c}{\partial x^{2}}-\frac{v}{n}\frac{\partial c}{\partial x}-R\gamma C$$

where *C* represents the concentration of pollutants at a distance *x* and time *t*, measured in mg/L; *x* denotes the distance along the direction of the groundwater flow, measured in m; *u* is the groundwater velocity, measured in m per second (m/s); *n* represents the effective porosity, which is a dimensionless quantity; *R* is the retardation factor; *γ* is the first-order decay rate, measured in seconds (s); and $D_{L}$ is the hydrodynamic longitudinal dispersion coefficient, measured in m per second (m/s).

The analytical solution for a continuously injecting point source is given by Equation (8) [25]:

(8) $$DAF=\frac{1}{2}erfc\left(\frac{x-ut}{2\sqrt{D_{L}t}}\right)+\frac{1}{2}e^{\frac{ux}{D_{L}}}erfc\left(\frac{x+ut}{2\sqrt{D_{L}t}}\right)$$

where *DAF* represents the groundwater dilution and attenuation factor, which is a dimensionless quantity; *x* denotes the distance along the direction of the groundwater flow, measured in m; *u* is the groundwater velocity, measured in m per day (m/d); $D_{L}$ is the longitudinal dispersion coefficient, measured in square meters per day (m^2^/d); and *erfc()* is the complementary error function.

### 2.4. Representation of Uncertainty and Risk

This study employs the Monte Carlo method to characterize the impact of parameter uncertainty on the results [8,17]. The relevant uncertain parameters are categorized into two types: those that exhibit significant regional variation, such as meteorological parameters like precipitation and evaporation; and those that show minor provincial differences or variations within the same province, such as groundwater conditions like the thickness of the vadose zone and aquifer. The risk is characterized from two dimensions: firstly, the probability of exceeding standards at exposure points, represented by the cumulative frequency distributions of predicted concentrations under uncertainty; and secondly, the use of the 95th percentile exposure point concentration to assess the multiple of exceedance. The Monte Carlo simulations were conducted using MATLAB (https://www.mathworks.com/products/matlab.html, accessed on 21 April 2025), with 10,000 iterations performed. The detailed values for the relevant parameters are provided in Table 1.

Based on annual precipitation, China can be divided into four types of dry–wet regions: humid, semi-humid, semi-arid, and arid. Specifically, humid regions have an annual precipitation generally above 800 mm, semi-humid regions have an annual precipitation between 400 mm and 800 mm, semi-arid regions have an annual precipitation between 200 mm and 400 mm, and arid regions have an annual precipitation below 200 mm. The precipitation in each region follows a uniform distribution.

For groundwater-related parameters: the unsaturated zone water content ratio, soil bulk density, groundwater mixing zone thickness, groundwater Darcy velocity, porosity, soil permeability, and other parameters adopt the recommended values from HJ 25.3-2019 [22].

For road-related parameters: the thickness of the subgrade layer is selected based on the domestic traffic highway standards, ranging from 0.3 m to 1.2 m; the width of the road, *W*, which is between 3.5 m and 30 m; and the horizontal distance from the road (exposure source) to the observation well, which is 100 m, with reference from the “Design Specifications for Highway Environmental Protection”, which specifies that the distance from the highway centerline to the water source should not be less than 100 m.

## 3. Results and Discussion

### 3.1. Release of Pollutants and Potential Environmental Risks

The leaching test results indicate that the leaching concentrations of the characteristic organics and Hg were below the detection limit, while the concentration of Cr(VI) was extremely low. In contrast, the leaching concentrations of the potentially toxic elements such as Ba, Co, Pb, Ni, As, and Mn were relatively high. Therefore, these six heavy metals are identified as the primary pollutants for subsequent analysis and evaluation.

The leaching concentrations of the six potentially toxic elements obtained using the NEN 7371 method are shown in Table 2. From Table 2, it can be seen that the maximum leaching concentrations of pollutants such as Ba, Pb, Ni, As, and Mn in the samples exceed their corresponding Class III groundwater limits. Further analysis reveals that the leaching concentrations of some pollutant components vary greatly among different samples. For example, the ratio of the maximum to minimum leaching concentration of Zn among different samples reaches 67 times, indicating a significant variability. Similarly, the ratios of the maximum to minimum leaching concentrations of Pb and Mn among different samples are also relatively large, reaching 15 and 34 times, respectively.

Considering that the value of the source strength concentration will affect the subsequent calculation of the groundwater risk assessment, the leaching concentrations of pollutant components are determined using the method for assigning uncertainty parameters. Statistical analysis is conducted based on the leaching concentrations of each pollutant in 100 samples, and the results show that their distribution follows a normal distribution. The distribution parameters (mean and standard deviation) are shown in Table 2.

**Figure 1:**
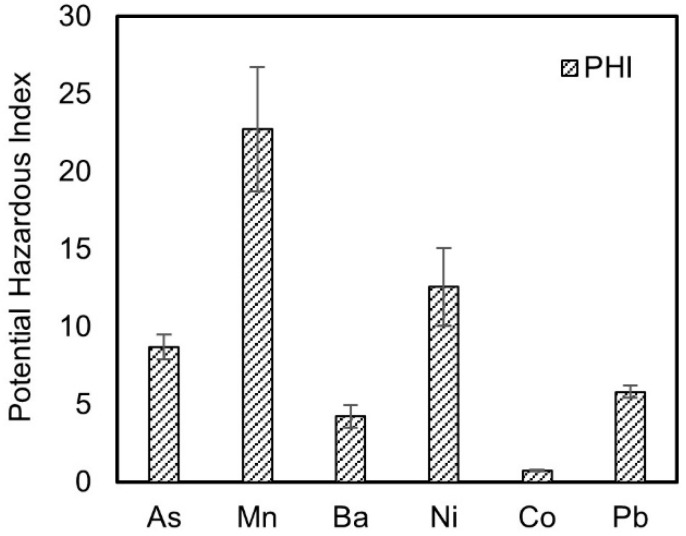
Potential hazard index of pollutant components.

The concentration of the pollutants at the exposure point is influenced by both the source strength concentration and the migration process in the groundwater, making the source strength concentration one of the determinant factors for the concentration in the observation wells. The ratio of this concentration to its corresponding environmental standard limit, known as the Potential Hazard Index (PHI), reflects the “potential” for the pollutant to exceed the standard [26]. The PHI values for the six pollutants, calculated using the mean leaching concentrations, are shown in Figure 1. Among the six inorganic pollutants, manganese has the highest PHI value of 22.7, while cobalt has the lowest of 0.74, differing by 31 times. This indicates that the potential for manganese to exceed the standard after migration and transformation in the groundwater is much greater than that of cobalt. According to the definition of the PHI, when the PHI of a pollutant is less than 1, it means that the measured leaching concentration is less than its corresponding Class III groundwater limit. Since the concentration of the pollutant in the observation wells during its migration in the groundwater is less than the source strength concentration, it can be predicted that cobalt poses little risk to the groundwater environment when solid waste is used as a subgrade cushion material. The five pollutants with PHI values greater than 1 are arsenic, manganese, barium, nickel, and lead, with specific values of 8.7, 22.7, 4.2, 12.6, and 5.8, respectively. Therefore, it can be predicted that the concentrations of these pollutants in the observation wells may follow the order: Mn > Ni > As > Pb > Ba.

### 3.2. Exposure Risk and Evolution of Utilizing Iron Tailings in Road Construction

**Figure 2:**
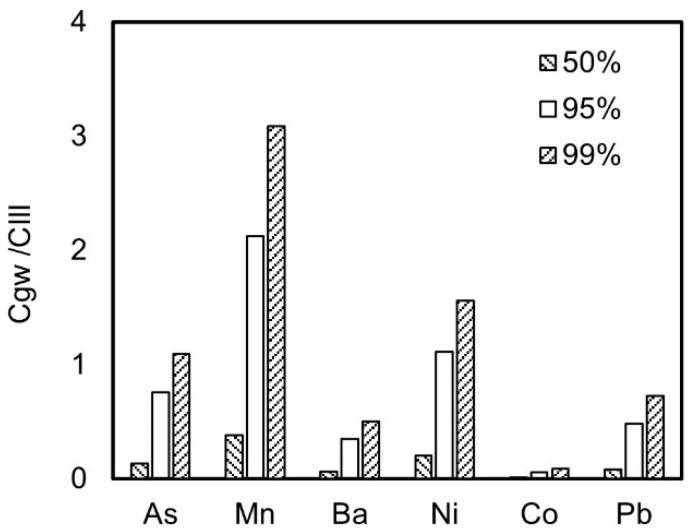
Comparison of the ratios of pollutant concentration to standard at the 50%, 95%, and 99% percentiles.

The upper limit of concentration control in the risk assessment is a crucial parameter for monitoring the risk of pollutants [25]. Currently, this concept is internationally defined as an estimate of the concentration at the pollutant exposure point. In China, the median or maximum concentration of pollutants is often used for risk assessments in solid waste resource utilization [7]. Therefore, this study compares the exposure concentrations in groundwater at different cumulative frequencies, as shown in Figure 2. From Figure 2, it can be seen that there are significant differences in the concentrations of the same pollutant at different cumulative frequencies. Taking nickel as an example, the concentrations at the 50%, 95%, and 99% cumulative frequencies are 0.2 mg/L, 1.1 mg/L, and 1.6 mg/L, respectively. The exposure concentration at the 50th percentile, which reflects the average level, is 18% of the value at the 95th percentile. The exposure concentration at the 99th percentile, which reflects an extremely unfavorable scenario, is 1.5 times the value at the 95th percentile. Obviously, if the median is taken as the exposure concentration, the risk value will be underestimated. Conversely, if the maximum value is taken, the risk assessment will be overly conservative, leading to excessive harmless disposals and increased resource and energy consumptions during the reuse process, which is not conducive to the synergistic enhancement of pollution reduction and carbon reduction. Therefore, we defined the upper limit of the 95% confidence interval as the exposure concentration.

**Figure 3:**
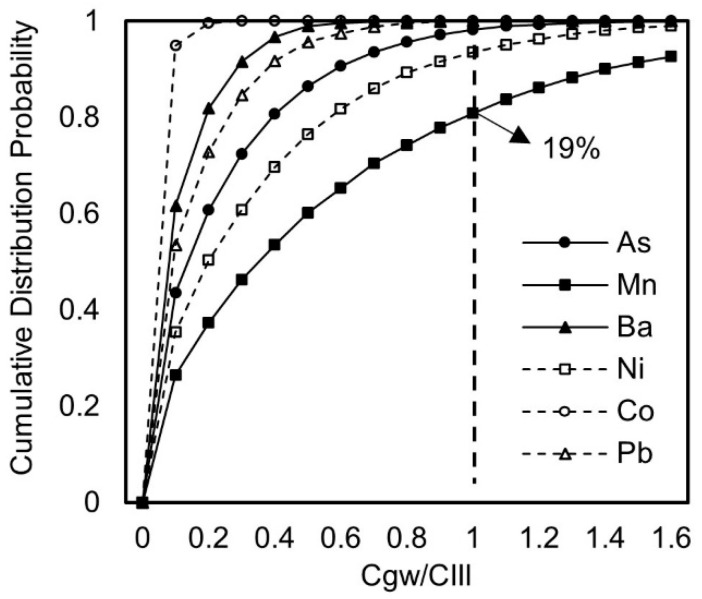
Cumulative distribution curves of the ratios of pollutant concentration to standard.

Using the Monte Carlo method, the cumulative frequency of the pollutant components obtained under conditions of uncertainty is shown in Figure 3. The cumulative frequency distribution of each pollutant is compared with its Class III groundwater quality limit to calculate its probability of exceeding the standard and the multiple of exceedance, obtaining the results of predicting the probability distribution and exposure concentration at the exposure point, using the NEN 7371 leaching concentration as the initial release concentration of the attenuation source model.

From Figure 3, it can be seen that the exposure concentrations of arsenic are all below their corresponding Class III groundwater limit of 0.01 mg/L, with the ratios of the concentration to the standard ranging from 0.0008 to 0.76, all below 1, indicating that the risk is controllable. Similarly, the exposure concentrations of barium, cobalt, and lead are all below their respective Class III groundwater limits, with the ratios of the concentration to the standard all below 1, indicating that the risks are within controllable ranges. However, there is a possibility that the ratios of the concentrations of manganese and nickel after migration and transformation in the groundwater to the Class III groundwater quality limits may be greater than 1. The probability of manganese exceeding the Class III groundwater quality limit is 19%, with an exposure concentration 2.1 times the limit. The probability of nickel exceeding the limit is 7%, with an exposure concentration 1.1 times the limit.

### 3.3. Regional Differences in Groundwater Pollution Risk from Utilizing Iron Tailings in Road Construction

Variations in hydrogeological and climatic conditions across different regions can affect the migration and dispersion processes of pollutant components [24]. Factors such as precipitation and net infiltration directly influence the release of harmful substances from roadbed fill materials [23]. This study focuses on the impact of precipitation differences in arid, semi-arid, sub-humid, and humid regions on the groundwater exposure risk associated with the use of iron tailings in road construction.

**Figure 4:**
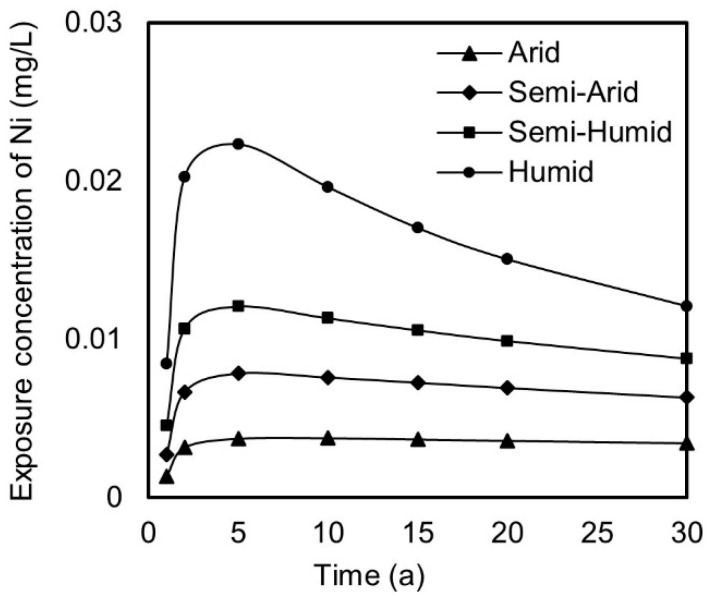
Trends of nickel concentration at exposure points over time (years).

Taking the heavy metal nickel as an example, Figure 4 shows the variability in nickel concentrations at exposure points over time in arid, semi-arid, sub-humid, and humid regions, simulated based on the model described in Section 2.3. From Figure 4, it can be observed that the nickel concentrations at exposure points in different regions initially increase over time, reach maximum values, and then gradually decrease or stabilize.

In humid regions with an annual precipitation exceeding 800 mm, pollutants in the iron tailing roadbed cushion are rapidly released into the groundwater with rainfall. The maximum nickel concentration at the exposure point reaches 0.253 mg/L in the second year, exceeding the Class III groundwater limit of 0.02 mg/L and posing a certain environmental risk. As the pollutants are gradually released, the concentration of pollutants released from the iron tailing roadbed cushion dilutes and attenuates over time, and the concentration at the exposure point decreases accordingly. In sub-humid regions with an annual precipitation between 400 mm and 800 mm, the maximum nickel concentration at the exposure point, which is 0.014 mg/L and does not exceed the Class III groundwater limit, is also reached in the second year. As the pollutants are gradually released, the concentration at the exposure point decreases slowly. In semi-arid and arid regions with even lower precipitation, the maximum nickel concentrations at the exposure points are lower, at 0.007 mg/L and 0.004 mg/L, respectively, and remain basically stable after reaching their maximum values.

The nickel concentration at the exposure point exhibits a two-stage change process. In the first stage, the pollutants diffuse spatially to the exposure point, resulting in an increase in pollutant concentration. In the second stage, due to the attenuation of source concentration and the dilution and diffusion of pollutants with the groundwater, the nickel exposure concentration at the exposure point decreases slowly. The higher annual precipitation in humid and sub-humid regions leads to faster attenuation of the source concentration in the iron tailing roadbed cushion, while the source concentration decays more slowly in arid and semi-arid regions. Therefore, regions with higher precipitations have higher maximum concentrations, while regions with lower precipitations have more sustained pollutant release. Studies by Ji et al. [27,28] have shown that, compared with arid/semi-arid regions, the environmental risk of the migration and diffusion of pollutants such as heavy metals is greater in humid/sub-humid regions. This indicates that, in the context of road construction using solid waste, exposure concentrations and environmental risks are positively correlated with precipitation and increase with increasing precipitation. This aligns with the model results of this study, as different precipitation levels in different regions lead to varying pollutant leaching amounts, suggesting that risks should be assessed reasonably and different control measures should be provided for different regions.

In addition, this study only considers the impact of meteorological parameters with the most significant regional differences on regional risk differences. Uniform distribution values are used for parameters related to groundwater migration and dispersion. Further research on the regional variability of groundwater-related parameters can be conducted in the future to refine regional difference assessments and management controls.

### 3.4. Study on Regionalized Differential Management and Control of Iron Tailings

**Figure 5:**
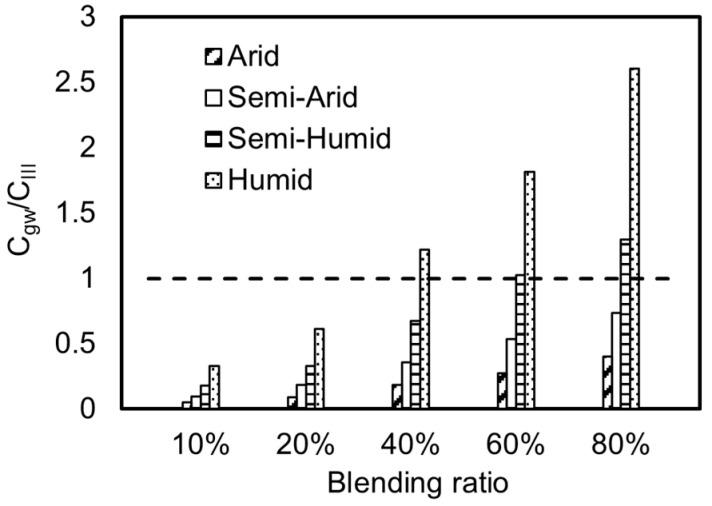
Comparison of the mixing ratios of iron tailings in arid, semi-arid, semi-humid, and humid regions.

When iron tailings are used in subgrade cushions, the risk of pollutant exposure can be controlled by adjusting the mixing ratio of iron tailings [9,26]. The maximum concentrations of pollutants at exposure points under different mixing ratios of iron tailings in arid, semi-arid, semi-humid, and humid regions were calculated. Through analysis, it was found that the pollutant factor most likely to cause groundwater exceedance is manganese. Therefore, manganese was selected as the research object to study the mixing ratios of iron tailings in different regions, as shown in Figure 5.

From the perspective of different regions, when iron tailings are used in subgrade cushions in arid or semi-arid regions, even if the mixing ratio of iron tailings is 100%, there is no possibility of exceeding the standard. Of course, considering that the annual precipitation is an average value, during the actual rainy season, there may be a possibility of instantaneous exposure concentrations exceeding the standard in arid and semi-arid regions. In semi-humid regions, the ratio of the manganese exposure concentration to the standard ranges from 0.18 to 1.75. When the mixing ratio reaches 60%, the manganese exposure concentration is 0.103 mg/L, slightly exceeding the Class III groundwater limit of 0.1 mg/L. In humid regions with the highest precipitation, the ratio of manganese to the standard ranges from 0.33 to 3.12, and the potential risk of exceeding the standard is significantly increased. Therefore, the mixing ratio of iron tailings should not exceed 35%.

Therefore, the environmental risk of iron tailing subgrade cushions is higher in humid regions, where the mixing ratio of iron tailings should not exceed 35%. In semi-humid regions, the mixing ratio should not exceed 60%. In arid and semi-arid regions, the mixing ratio of iron tailings can be appropriately increased.

While this study provides valuable insights into the environmental risks associated with utilizing iron tailings in road construction, there are several limitations that warrant further improvements, particularly with respect to climate change and extreme weather conditions. The model assumes that certain parameters, such as meteorological variables, follow uniform distributions, which may not adequately capture the spatial and temporal variability under changing climatic conditions, especially in regions prone to extreme rainfall events, potentially affecting the reliability of predictions under future climate scenarios. Additionally, in assessing the pollutant migration and dilution processes, the current study adopts simplified representations that neglect the long-term impacts of adsorption and chemical reactions—factors that may play a critical role during intense precipitation events where increased runoff and soil saturation could enhance contaminant mobilization—leading to the potential underestimation or overestimation of pollutant behavior under extreme climatic conditions.

## 4. Conclusions

This study provides an in-depth analysis of the environmental risks associated with the road application of solid waste iron tailings. Using Monte Carlo simulations, the environmental risks of utilizing iron tailings in road construction in arid, semi-arid, semi-humid, and humid regions were assessed. The results indicated that manganese and nickel concentrations may exceed Class III groundwater quality limits after migration and transformation in the groundwater. Specifically, the probability of manganese exceeding the limit is 23%, with an exposure concentration that can reach 2.1 times the limit; the probability of nickel exceeding the limit is 7%, with an exposure concentration that is 1.1 times the limit. The study revealed that as precipitation increases, the risk of leaching of potentially toxic elements and other pollutants from iron tailings also increases, but this risk can be controlled by adjusting the mixing ratio of iron tailings in the subgrade cushion. In humid regions, the environmental risk associated with iron tailing subgrade cushions is higher, and the mixing ratio of iron tailings should not exceed 35%. In semi-humid regions, the mixing ratio should not exceed 60%, while in arid and semi-arid regions, the mixing ratio of iron tailings can be appropriately increased. The study emphasizes that traditional methods not only underestimate the release potential and peak risk of pollutants but also neglect the regional characteristics of risk. Therefore, there is an urgent need for national-level improvements in risk assessment and management standards for solid waste resource utilization and to implement regionally differentiated management strategies for hazardous substances. Looking ahead, future research should incorporate the impacts of climate change into the environmental risk assessment framework. With the increasing frequency and intensity of extreme weather events—particularly rainfall—it is essential to consider how changing precipitation patterns may affect the long-term behavior of contaminants derived from iron tailings. In addition to annual precipitation, parameters such as “maximum daily precipitation” and “absolute largest daily precipitation” should be integrated into the Monte Carlo model to better capture the influence of short-term, high-intensity rainfall events on pollutant leaching. This is especially critical in arid and semi-arid areas, where even rare but intense rainfall can significantly enhance contaminant mobilization.

## Figures and Tables

**Table 1:** Range of distribution for uncertainty parameters.

Parameters	Symbol	Unit	Input Value
Infiltration coefficient	*α*	Dimensionless	N (0.2, 0.05)
Subgrade layer thickness	*d*	m	U (0.3, 1.2)
Solid waste volume fraction	*f*	Dimensionless	U (0.1, 0.5)
Subgrade material density	*ρ*	kg/L	N (2, 0.2)
Soil bulk density	*ρ_b_*	kg/L	1.5
Water content ratio of unsaturated zone	*θ_ws_*	Dimensionless	0.3
Groundwater Darcy velocity	*U_gw_*	m/a	25
Groundwater mixing zone thickness	*σ_gw_*	m	N (2, 0.082)
Soil permeability	*I*	m/a	0.3
Road width	*W*	m	U (3.5, 30)
Hydraulic conductivity	*K*	m/d	U (10, 50)
Hydraulic gradient	*i*	‰	U (3, 6)
Porosity	*n*	%	U (30, 50)
Longitudinal dispersion coefficient	*DL*	m^2^/d	U (5, 10)

**Table 2:** Statistical summary of leaching concentrations of pollutants determined by the NEN 7371 method (mg L^−1^).

Pollutant	As	Mn	Ba	Ni	Co	Pb
Mean	0.040	1.860	2.600	0.080	0.080	0.050
Standard Deviation	0.005	0.600	0.300	0.010	0.006	0.01
Minimum	0.030	0.253	1.691	0.057	0.057	0.025
Maximum	0.053	3.325	3.228	0.097	0.097	0.091
Class III Limit	0.01	0.1	0.7	0.02	0.05	0.01

## Data Availability

The data that support the findings of this study are available from the corresponding author upon reasonable request.
